# Factors for Enhancement of Intracranial Atherosclerosis in High Resolution Vessel Wall MRI in Ischemic Stroke Patients

**DOI:** 10.3389/fneur.2020.00580

**Published:** 2020-06-26

**Authors:** Na-Eun Woo, Han Kyu Na, Ji Hoe Heo, Hyo Suk Nam, Jin Kyo Choi, Sung Soo Ahn, Hyun Seok Choi, Seung-Koo Lee, Hye Sun Lee, Jihoon Cha, Young Dae Kim

**Affiliations:** ^1^Department of Neurology, Yonsei University College of Medicine, Seoul, South Korea; ^2^Department of Radiology, Yonsei University College of Medicine, Seoul, South Korea; ^3^Biostatistics Collaboration Unit, Department of Research Affairs, Yonsei University College of Medicine, Seoul, South Korea

**Keywords:** stroke, atherosclerosis, dyslipidemia, vessel wall MRI, high resolution MRI

## Abstract

**Introduction:** High resolution vessel wall MRI (VW-MRI) has enabled to characterize intracranial atherosclerosis (ICAS). We studied to identify the factors for enhancement of ICAS in VW-MRI in patients with acute ischemic stroke.

**Methods:** Consecutive patients with acute ischemic stroke or TIA who underwent VW-MRI between January 2017 and December 2017 were included. Enhancement on VW-MRI was defined as an increase in intensity on contrast-enhanced T1-weighted sequence. We compared the clinical and the radiologic findings between patients with wall enhancement and those without wall enhancement.

**Results:** Of the 48 patients with ICAS, 28 patients revealed enhancement on VW-MRI. Patients with enhancement were more likely to have severe stenotic lesions and higher levels of total cholesterol, triglycerides, low-density cholesterol, Apo (b), and Apo (b)/Apo (a) lipoprotein ratio (*p* < 0.05). Multivariable analysis demonstrated that total cholesterol (OR: 5.378, 95% CI, 1.779–16.263), triglycerides (OR: 3.362, 95% CI, 1.008–11.209), low density lipoprotein cholesterol (OR: 4.226, 95% CI, 1.264–14.126), Apo (b) lipoprotein (OR: 3639.641, 95% CI, 17.854–741954.943) levels, and Apo (b)/Apo (a) lipoprotein ratio (OR, 65.514; 95% CI, 1.131–3680.239) were independently associated with enhancement of ICAS. High-density lipoprotein cholesterol and Apo (a) lipoprotein levels were not significantly different between the patients with wall enhancement and those without wall enhancement.

**Conclusions:** The presence and severity of enhancement of ICAS was significantly associated with dyslipidemic conditions. These results suggest that strict lipid modification should be achieved for the management of ICAS.

## Introduction

Ischemic stroke is a major cause of mortality and morbidity worldwide (1). Atherosclerosis, in particular, intracranial atherosclerosis (ICAS), is recognized as one of the most common etiological factors of ischemic stroke accounting for 30–50% of the ischemic cerebrovascular events (2). Moreover, ischemic stroke attributable to ICAS is known to be associated with an increased risk of poor functional outcomes after stroke, cognitive impairment, and vascular death (3). Precise in vivo characterization of ICAS is crucial, as this knowledge enables the clinicians to better stratify the stroke risk for individual patients and to plan therapeutic strategies accordingly.

Conventionally, clinicians have focused primarily on detection and estimation of the degree of luminal stenosis by using luminography techniques such as digital subtraction angiography or magnetic resonance angiography (MRA). However, luminography alone is insufficient to fully determine the histopathologic composition and instability of the plaque (4, 5). Currently, the advent of high resolution vessel wall magnetic resonance imaging (VW-MRI) has enabled clinicians to differentiate among various intracranial vasculopathies and to characterize the vulnerability of ICAS plaque, which is thought to be the culprit lesion of ischemic events, and the degree of luminal stenosis (6–9). Among the VW-MRI findings, contrast enhancement of the ICAS plaque is increasingly being reported as a reliable marker of plaque vulnerability. It also tends to be predictive of future cerebrovascular events (4, 10, 11). However, the factors for the presence or extent of arterial wall enhancement on VW-MRI are not fully understood (12, 13).

In the present study, we aimed to identify the factors for the enhancement of ICAS in VW-MRI in patients with acute ischemic stroke.

## Methods

### Patients

We performed a retrospective analysis of all the patients with ischemic stroke admitted to Severance Stroke Center for the treatment of cerebral infarction or transient ischemic attack within seven days after the onset of symptoms between January 2017 and December 2017. At the time of admission, all the patients were thoroughly evaluated to record the demographic data, medical history, clinical manifestations, and vascular risk factors (14). All patients underwent brain computed tomography and/or magnetic resonance imaging with cerebral angiographic studies, standard blood tests, and 12-lead electrocardiography. Cerebral angiographic studies including digital subtraction angiography, MRA, or computed tomography angiography covering intracranial arterial beds, the proximal carotid portion, and vertebral orifice were performed. Most of the patients were admitted to a stroke unit and were monitored continuously with electrocardiography during their stay (average 4.9 days). Holter monitoring was also performed if a cardiac embolism was suspected based on the infarction pattern, patient's age, or previous cardiac history. Transesophageal echocardiography was included in the routine examination, unless it could not be performed due to patient's condition or the failure to obtain an informed consent.

Among the patients admitted during the study period, we included patients who underwent VW-MRI during hospitalization. Patients were excluded if they had (1) at least one of the high risk potential cardiac sources of embolism based on Trial of ORG 10272 in Acute Stroke Treatment classification (15), (2) nonatherosclerotic vasculopathies such as aortic dissections, vasospasm, vasculitis, or moyamoya disease, and (3) poor quality of imaging data. The present study was approved by the institutional review board of Severance Hospital, Yonsei University Health System. Informed consent was waived due to the retrospective nature of the study.

### MR Imaging Analysis

All the scans were performed using a Philips Ingenia 3.0T Cx MR scanner (Philips Medical systems, Best, The Netherlands) with a 32-channel head coil. TOF-MRA scan was primarily used to find the location and the degree of stenosis in ICAS. The neuroradiologist (J.C.) selected the site of evaluation and the appropriate protocol based on the clinical presentation and TOF-MRA findings. For vessel wall evaluation, high resolution 3-dimensional proton-density (3D-PD), pre/post-contrast enhanced T1WI with black-blood techniques (3D turbo spin echo sequence with improved motion-sensitized driven-equilibrium preparation) (T1-BB pre/post) were performed using the following parameters: (1) TOF-MRA: TR−19 ms, TE−3.5 ms, FOV−210 × 210 mm, matrix size−640 × 330, slice thickness−1.2 mm (interpolated to 0.6 mm), 9.6 cm coverage on axial plane which covered all intracranial major arteries with maximum intensity projection reconstruction, and acquisition time−5 min 10 sec. (2) 3D-PD: TR−1,800 ms, TE−37.4 ms, FOV−180 × 180 mm, matrix size−384 × 384 mm, slice thickness−0.5 mm (interpolated to 0.3 mm), 4 cm coverage on coronal plane with axial and sagittal reconstruction, and acquisition time−9 min 37 sec. (3) T1-BB pre/post: TR−650 ms, TE−34.8 ms, FOV−180 × 180 mm, matrix size−304 × 304 mm, slice thickness−0.6 mm (interpolated to 0.3 mm), 4 cm coverage on coronal plane with axial and sagittal reconstruction, and acquisition time−8 min 21 s.

### Clinical and Imaging Variables

We collected the patient data on vascular risk factors such as hypertension, diabetes, dyslipidemia, body mass index (kg/m^2^), metabolic syndrome according to National Cholesterol Education Program Expert Panel and Adult Treatment Panel III criteria, current smoking, prior history of ischemic heart disease, ischemic stroke, or peripheral artery occlusive disease, and prior medication status. We defined hypertension when systolic blood pressure was higher than 140 mmHg or patients prescribed medication, and also defined diabetes or dyslipidemia when patients had medication or their lab findings were met the diagnostic criteria. We also investigated the laboratory data including complete blood count, blood urea nitrogen, creatinine, serum protein and albumin level, fasting glucose, HbA1c, erythrocyte sedimentation ratio, C-reactive protein, D-dimer, creatinine kinase, fibrinogen levels, and lipid profiles (total cholesterol, triglycerides, high-density lipoprotein cholesterol [HDL-C], low-density lipoprotein cholesterol [LDL-C], non-HDL cholesterol, Apo [b] lipoprotein, Apo [a] lipoprotein, and Apo [b]/Apo [a] ratio). The lipid profile was drawn from the antecubital vein 8 hours after an overnight fast. All blood tests were performed at the Department of Laboratory Medicine, Yonsei University College of Medicine. Total cholesterol, triglyceride, HDL-C, and LDL-C was Enzymatic methods using Atellica CH 930 Analyzer (Siemens Healthcare Diagnostics, Marburg, Germany) and Apo (b) and Apo (a) lipoprotien was done by immunoturbidimetric assay using Cobas c702 (Roche Diagnostics, Switzerland). Stroke severity was assessed using the National Institutes of Health Stroke Scale.

We investigated the enhancement of the culprit intracranial plaque, which was the lesion within the same vascular territory as that of the stroke. In the case of multiple lesions, we decided culprit lesion within the same vascular territory as that of the major infarctions. When a patient had a lesion with the enhancement, the degree of enhancement was classified as mild to moderate or strong, based on comparison with the signal intensity of the pituitary stalk. In addition, we collected the data on the presence of high T1 signal of plaque, enhancement type (eccentric or non-eccentric) and reconstruction index (the ratio of the outer wall diameter at the lesion site to that at the contralateral normal site) among patients with enhancement. Based on the degree of stenosis of ICAS, we divided patients into four groups including no stenosis, mild to moderate stenosis (<50%), severe stenosis (≥ 50%), and occlusion. For categorizing the severity of arterial stenotic lesions, the degree of stenosis was measured in culprit arterial segment based on the Warfarin vs. Aspirin for Symptomatic Intracranial Disease method (16). The radiologic findings were interpreted by a radiologist and a neurologist (J.C and N.E.W.) who were blinded to clinical information. In case of a discrepancy, the final decision was made by a third reviewer (Y.D.K).

### Statistical Analysis

Statistical analyses were performed using the Windows SPSS package (version 24.0, IBM Corp., Armonk, NY, USA) and R version 3.4.2 (http://www.R-project.org). The analyses were performed using the independent t-test, one way analysis of variance, Mann-Whitney *U* test, or the Kruskal-Wallis test for the continuous variables and the χ2 test or the Fisher's exact test for categorical variables, as appropriate. Spearman correlation analysis was used to investigate the relationship between reconstruction index and continuous variables. When we investigated the trend of lipid levels by the degree of enhancement, one way analysis of variance using trend contrasts was used. To determine the independent factors for the enhancement of ICAS on VW-MRI, multiple logistic regression analysis was used with adjustment for potential confounders with *p* < 0.05 in the univariable analysis. A two-tailed p-value less than 0.05 was considered statistically significant.

## Results

Out of the 79 patients who had acute ischemic stroke and who underwent the VW-MRI during the study period, we included 48 patients after excluding 3 patients with high risk potential cardiac sources of embolism, 26 patients with nonatherosclerotic vasculopathies, and 2 patients with poor quality of imaging data.

The baseline characteristics are presented in Table 1. The mean age was 57.0 years and 64.6% (31/48) of the patients were male. Twenty patients had no arterial lesion with enhancement, while 28 patients showed enhancement (8 with mild to moderate enhancement and 20 with strong enhancement) on VW-MRI. The location of the arterial segments with enhancement was middle cerebral artery in 16 patients, vertebral and basilar artery in 8 patients, and distal internal carotid artery in 2 patients.

**Table 1:** Baseline characteristics of patients and comparison between patients with enhancement and patients without enhancement.

	**Total (*n* = 48)**	**Wall enhancement **(−)** (*n* = 20)**	**Wall enhancement (+) (*n* = 28)**	***p***
Age, y	57.0 ± 16.4	53.5 ± 18.8	59.5 ± 14.2	0.21
Male sex	31 (64.6)	13 (65.0)	18 (64.3)	0.86
Body mass index, kg/m^2^	24.8 ± 3.4	24.6 ± 4.2	24.9 ± 2.9	0.81
Hypertension	28 (58.3)	9 (45.0)	19 (76.9)	0.11
Diabetes	14 (29.2)	7 (35.0)	7 (25.0)	0.46
Hyperlipidemia	11 (22.9)	2 (10.0)	9 (32.1)	0.09
Metabolic syndrome	1 (2.1)	0 (0.0)	1 (3.6)	1
Smoker	25 (52.1)	10 (50.0)	15 (53.6)	0.81
Previous ischemic heart disease	13 (27.1)	9 (45.0)	4 (14.3)	0.02
Peripheral arterial occlusive diseases	2 (4.2)	1 (5.0)	1 (3.6)	1
Previous ischemic stroke	13 (27.1)	8 (40.0)	5 (17.9)	0.09
Prior statin use		10 (50.0)	5 (17.9)	0.012
Cerebral artery stenosis				<.001
No stenosis	8 (16.7)	8 (40.0)	0 (0.0)	
Mild to moderate stenosis (<50%)	13 (27.1)	1 (5.0)	12 (49.3)	
Severe stenosis (≥ 50%)	19 (39.6)	4 (20.0)	15 (53.6)	
Occlusion	8 (16.7)	7 (35.0)	1 (3.6)	
Laboratory findings				
Total cholesterol, mmol/L	4.7 ± 1.1	4.1 ± 1.2	5.2 ± 0.8	<.001
Triglyceride, mmol/L	1.8 ± 1.4	1.3 ± 0.6	2.1 ± 1.6	0.01
High density lipoprotein cholesterol, mmol/L	1.2 ± 0.3	1.1 ± 0.3	1.2 ± 0.3	0.23
Low density lipoprotein cholesterol, mmol/L	2.9 ± 0.9	2.4 ± 0.9	3.2 ± 0.8	0.001
Non-HDL cholesterol, mmol/L	3.5 ± 1.0	2.9 ± 1.1	4.0 ± 0.8	<0.001
Apo (a) lipoprotein, g/L	1.4 ± 0.3	1.3 ± 0.2	1.4 ± 0.3	0.17
Apo (b) lipoprotein, g/L	1.1 ± 0.3	0.9 ± 0.3	1.2 ± 0.2	<.001
Apo (b)/Apo (a) ratio	0.8 ± 0.2	0.7 ± 0.2	0.9 ± 0.2	0.003
Blood urea nitrogen, mmol/L	5.8 ± 2.1	5.7 ± 1.9	5.9 ± 2.2	0.86
Creatinine, μmol/L	69.1 ± 22.3	71.3 ± 15	67.5 ± 26.5	0.58
Albumin, g/L	42.6 ± 3.2	42.8 ± 3.2	42.4 ± 3.2	0.73
Protein level, g/L	69.8 ± 5.2	69.4 ± 5.8	70 ± 4.8	0.66
Creatine kinase, μkat/L	2.3 ± 2.1	3 ± 2.8	1.7 ± 1.1	0.06
C-reactive protein, nmol/L	44.7 ± 169.5	74.6 ± 261.3	23.6 ± 37.2	0.32
D-dimer, nmol/L	1167.5 ± 1468.4	1616 ± 2008.7	847.2 ± 811.5	0.12
White blood cell count, 10^9^/L	0.008 ± 0.002	0.008 ± 0.003	0.008 ± 0.002	0.82
Hemoglobin, g/L	147.6 ± 18.8	144.3 ± 18.3	150 ± 19.1	0.31
Platelet count	229.4 ± 52.6	215.9 ± 69.3	239.1 ± 34.8	0.18
HbA1c	0.06± 0.01	0.06 ± 0.01	0.06 ± 0.01	0.26
Fasting glucose, mmol/L	8.1 ± 3.7	7.6 ± 3.3	8.4 ± 4	0.45
Erythrocyte sedimentation ratio, mm/h	18.3 ± 15.3	15.6 ± 14.8	20 ± 15.7	0.35
Fibrinogen level, μmol/L	8.8 ± 2.3	8.2 ± 1.7	9.3 ± 2.6	0.11

*Data are presented as numbers (%) or mean ± standard deviation*.

**Figure 1:**
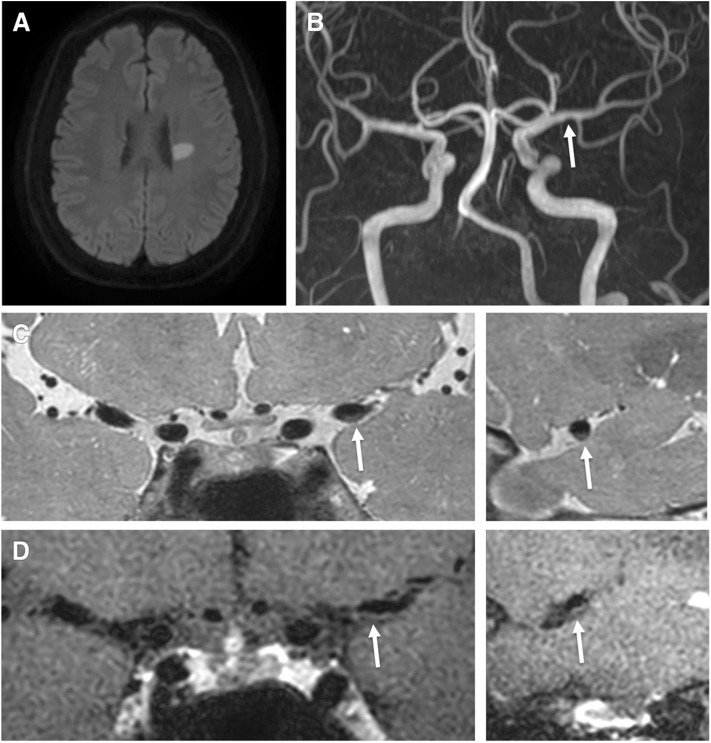
A 62-year-old male patient presented with right hemiparesis with low-density cholesterol, total cholesterol, and Apo (b) levels of 67 mmol/L, 113 mmol/L, 75.5 g/L, respectively. **(A)** Axial DWI shows a high signal intensity lesion in the left corona radiata region. **(B)** TOF-MRA shows mild stenosis in the left middle cerebral artery (proximal M1, arrow). **(C)** Pre-contrast 3-dimensional proton-density black-blood magnetic resonance images. **(D)** The post-contrast images (left: coronal plane, right: sagittal plane) show eccentric wall thickening (arrow) without enhancement.

**Figure 2:**
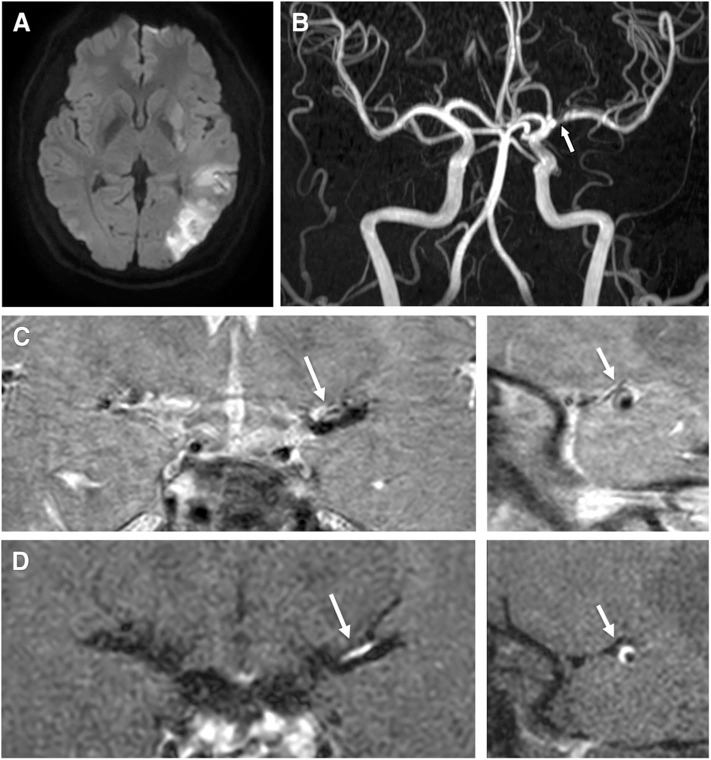
A 40-year-old male patient presented with right hemiparesis and aphasia with low-density cholesterol, total cholesterol, and Apo (b) levels of 178 mmol/L, 225 mmol/L, and 137 g/L, respectively. **(A)** Axial DWI shows a high signal intensity lesion in the left parietal region. **(B)** TOF-MRA shows severe stenosis in the left middle cerebral artery (proximal M1, arrow). **(C)** Pre-contrast 3-dimensional proton-density black-blood magnetic resonance images. **(D)** Post-contrast images (left: coronal plane, right: sagittal plane) show eccentric wall thickening (arrow) with enhancement.

**Figure 3:**
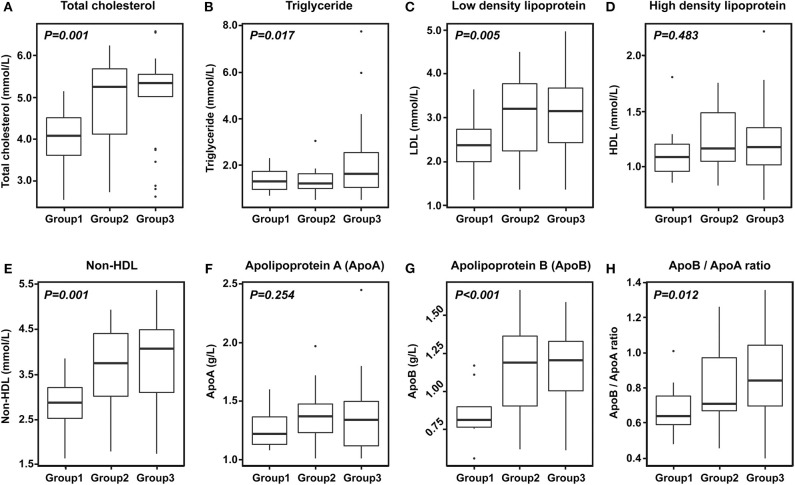
Degree of enhancement of intracranial atherosclerosis according to lipid levels. **(A)** Total cholesterol. **(B)** Triglyceride. **(C)** Low density lipoprotein cholesterol. **(D)** High density lipoprotein cholesterol. **(E)** Non-HDL cholesterol. **(F)** Apolipoprotein A. **(G)** Apolipoprotein B. **(H)** ApoB/ApoA ratio. Group 1: no enhancement, Group 2: mild to moderate enhancement, Group 3: strong enhancement.

Patients with enhancement were more likely to have severe stenotic lesions and higher levels of total cholesterol, triglycerides, LDL-C, non-HDL cholesterol, Apo (b), and Apo (b)/Apo (a) lipoprotein ratio (p < .05) (Table 1, Figures 1, 2). Moreover, patients with enhancement were less likely to have a prior history of ischemic heart disease or a history of statin use. After investigating the relationship between the severity of enhancement and the lipid indices, a gradual increase in the lipid level was noted with increase in the degree of enhancement (Figure 3). However, there was no significant difference in HDL-C and Apo (a) lipoprotein levels with respect to enhancement of ICAS.

Multivariable analysis adjusting prior history of ischemic heart disease, prior statin use, and the presence of severe steno-occlusive lesions demonstrated that total cholesterol (OR: 5.378, 95% CI, 1.779–16.263), triglyceride (OR: 3.362, 95% CI, 1.008–11.209), LDL-C (OR: 4.226, 95% CI, 1.264–14.126), non-HDL cholesterol (OR: 4.928, 95% CI, 1.519–15.982), Apo (b) lipoprotein (OR: 3639.641, 95% CI, 17.854–741954.943) levels, and Apo (b)/Apo (a) lipoprotein ratio (OR: 65.514, 95% CI, 1.131–3680.239) were independently associated with enhancement of ICAS (Table 2). However, HDL-C and Apo (a) lipoprotein levels were not significantly different between patients with enhancement and those without enhancement (Table 2).

**Table 2:** Factors for enhancement of ICAS on VW-MRI.

	**Univariable analysis**		Multivariable analysis^*^	
	**Odds ratio (95% CI)**	***p* value**	**Odds ratio (95% CI)**	***p* value**
Total cholesterol, mmol/L	3.123 (1.544–6.317)	0.002	5.378 (1.779–16.263)	0.003
Triglyceride, mmol/L	2.515 (1.031–6.137)	0.04	3.362 (1.008–11.209)	0.05
High density lipoprotein cholesterol, mmol/L	3.672 (0.444–30.336)	0.227	8.172 (0.795–83.979)	0.08
Low density lipoprotein cholesterol, mmol/L	3.236 (1.458–7.179)	0.004	4.226 (1.264–14.126)	0.02
Non-HDL cholesterol, mmol/L	3.164 (1.525–6.562)	0.002	4.928 (1.519–15.982)	0.008
Apo (a) lipoprotein, g/L	5.377 (0.470−61.507)	0.18	9.862 (0.768–126.555)	0.08
Apo (b) lipoprotein, g/L	181.794 (9.412–3511.318)	0.001	3639.641 (17.854–741954.943)	0.003
Apo (b) lipoprotein/Apo (a) lipoprotein	86.707 (3.301–2277.377)	0.007	64.514 (1.131–3680.239)	0.04

* *After adjusting coronary artery occlusive disease, prior statin use, and severe cerebral stenotic lesion*.

The presence of enhancement tended to be related to the lipid profiles regardless of age (> 57 years [median age of our study population] vs. ≤ 57 years) or sex (Supplementary Table 1), although several results related with triglyceride, non-HDL, Apo (b)/Apo (a) ratio did not reach statistical significance. When we investigated which factor affected high T1 signal, enhancement type, or reconstruction index, the presence of high T1 signal intensity was associated with platelet count (*p* = 0.02) and severe steno-occlusive lesion (*p* = 0.024), while eccentric enhancement was related with severe stenosis (*p* = 0.021) and blood urea nitrogen (*p* = 0.026). However, these findings of VW-MRI were not associated with the dyslipidemic condition (Supplementary Tables 2, 3).

## Discussion

In the present study, we aimed to identify the potential factors for enhancement of the culprit plaque of ICAS. We observed that the presence of intracranial arterial culprit plaque with enhancement on VW-MRI was associated with higher levels of total cholesterol, triglycerides, LDL-C, non-HDL cholesterol, Apo (b) lipoprotein, and Apo (b)/Apo (a) lipoprotein ratio (the so called “bad types of cholesterol”). The levels of HDL-C and Apo (a) lipoprotein were not significantly different between patients with enhancement and those without enhancement. These associations remained statistically significant even after adjusting for potential confounders. These findings indicated that the enhancement of ICAS was independently associated with dyslipidemic conditions. Moreover, there was a gradual increase in these lipid indices with increase in the degree of enhancement, indicating that the degree of ICAS enhancement is dependent on the severity of dyslipidemia.

Accumulating evidence suggests that luminal stenosis alone is not sufficient to fully explain the characteristics of ICAS (6–8) in terms of the composition or the activity of the culprit plaque (4, 5). In the early stages of atherosclerosis, the accumulation of plaque burden could take place without significant luminal narrowing. Such an accumulation is not detectable by conventional luminography techniques. However, this plaque can cause ischemic stroke when it is highly vulnerable (17, 18). This finding has led researchers to expand their focus beyond stenosis and toward the plaque morphology or plaque instability in an attempt to identify more accurate imaging markers to predict the risk of stroke related to ICAS (17). VW-MRI can be a promising technique for reliably imaging the intracranial arterial wall with superior soft tissue contrast and spatial resolution (9).

Recent studies have suggested that vessel wall enhancement on VW-MRI may be indicative of vulnerable or symptomatic plaque, as it is commonly associated with multiple embolic infarctions and has approximately four times higher recurrent stroke risk (30% annual stroke risk) in future (4, 10, 11). However, the factors for enhancement of the culprit plaque of ICAS in patients with recent symptomatic ischemic stroke have not yet been thoroughly investigated. Some reports have claimed that the risk factors for atherosclerotic process such as age, hypertension, or dyslipidemia may induce development and proliferation of the vasa vasorum (19), which subsequently results in the ICAS related arterial wall enhancement (20–22). Therefore, we aimed to evaluate the potential association between ICAS plaque enhancement and vascular risk factors including dyslipidemic conditions. We hypothesized that the enhancing lesion on VW-MRI is suggestive of plaque hemorrhage, neovascularization, or inflammation (23), and the lipid metabolism may play an important role in this process (24). Our analysis showed that triglycerides, LDL-C, non-HDL cholesterol, and Apo (b) lipoprotein (the so-called “bad cholesterols”) were independent and significant risk factors for plaque enhancement on VW-MRI, while the other risk factors for atherosclerosis were not associated with the plaque enhancement of ICAS. Moreover, we found a dose-dependent relationship between lipid levels and the degree of enhancement, indicating that dyslipidemic conditions play an important role in ICAS and subsequent stroke.

Contrast enhancement of atherosclerotic plaque was suggestive of a rich vascular supply into the plaque and enhanced endothelial permeability. In previous MRI studies of histopathologically confirmed cervical artery atherosclerosis, the plaque enhancement was significantly associated with the loose fibrous cap, neovascularization in the adventitia of the artery, macrophages, and increased endothelial permeability (25–27). Compared with extracranial atherosclerosis, investigating the ICAS plaque enhancement using vessel-wall imaging is quite challenging, mainly due to the difficulty of performing histologic validation in ICAS. Therefore, the pathophysiology, composition, and vulnerability of the ICAS plaque behind the enhancement are merely based on the assumption of imaging features of ICAS. Moreover, the lessons learned from the extracranial atherosclerosis cannot be directly extrapolated to ICAS, as the histological features of intracranial vessels are different from those of the carotid arteries in that it has ICAS has denser internal elastic lamina and less abundant adventitia with external elastic lamina (28). However, in the process of ICAS plaque formation, development and proliferation of the vasa vasorum related to aging and atherosclerotic process could also occur, subsequently leading to arterial wall enhancement, as shown in carotid plaques (19, 29).

In the present study, HDL and Apo (a) lipoprotein were not related with the enhancement of ICAS. Some studies investigating the association between lipid profiles and ICAS in the Asian population suggested that the burden of ICAS could be affected more by LDL-C, total cholesterol, non-HDL cholesterol, or Apo (b)/Apo (a) lipoprotein ratio rather than HDL-C (30–33). High levels of total cholesterol or high Apo (b)/Apo (a) lipoprotein ratio were reported to be associated with progression of ICAS (34). Our results were consistent with these previous findings, which suggested that the vulnerability of ICAS could be affected more by non-HDL cholesterol or Apo (b) lipoprotein, than HDL or Apo (a) lipoprotein.

Considering the relationship between the enhancement of ICAS and higher lipid levels, lowering the lipid levels by means of intensive statin therapy could decrease the chance of vulnerable plaque development. Previous studies have suggested that intensive statin therapy tends to be associated with lower volume of ICAS enhancement and thus, may be helpful in preventing the embolic occlusion (20). Given the association between the enhancement of ICAS and future risk for recurrent stroke, our findings suggest the need for intensive lipid lowering therapy in stroke management. There is no target level specific to ICAS with history of stroke. However, lipid-lowering therapy using statin with or without ezetimibe with the target of LDL-C <70 mg/dL or non-HDL cholesterol <100 mg/dL may be a viable option. It is the target recommended for ‘patients with atherosclerotic cardiovascular disease with very high risk' according to recent guidelines for management of blood cholesterol (35). Further investigations should focus on the optimal cholesterol level that may reduce the burden of ICAS.

The present study has several limitations. It was a cross-sectional study, which makes it difficult to establish the causal association. The sample size was small, since we included patients who were admitted for symptomatic acute ischemic stroke and underwent VW-MRI at a single center. Enhancement of arterial wall could also be associated with other conditions mimicking ICAS such as age-related vasa vasorum in the intracranial arteries, normal enhancement of veins, and slow flow artifacts (9, 19). However, efforts were made in the present study to distinguish ICAS with its pitfalls based on the pattern and the topographic distribution of vessel wall enhancement. We applied the black-blood technique to distinguish slow flow artifacts from arterial wall thickening. Hence, we believe that the possibility of including pathologies other than ICAS was small. Lastly, because the lipid profiles could be changed during acute stroke period, the pattern of enhancement could be changed along with the changing lipid profiles. However, we could not investigate the dynamic change of enhancement of VW MRI in this study.

The present study demonstrated that the presence and severity of enhancement of ICAS, especially the culprit lesion, tends to be associated with dyslipidemic conditions. Our findings suggest that strict lipid modification may be effective in the management of ICAS. Further longitudinal studies concentrating on the change in the ICAS burden after lipid modification therapy are required to fully address this issue.

## Data Availability Statement

The raw data supporting the conclusions of this article will be made available by the authors, without undue reservation.

## Ethics Statement

The studies involving human participants were reviewed and approved by The present study was approved by the institutional review board of Severance Hospital, Yonsei University Health System (approval no. 4-2019-0532). Written informed consent for participation was not required for this study in accordance with the national legislation and the institutional requirements.

## Author Contributions

N-EW: acquisition of data, analysis and interpretation of data, writing of original draft. HKN, JH, HSN, JKC, SA, HC, and S-KL: acquisition of data and interpretation of data. HL: analysis and interpretation of data. JC, study concept and design, analysis and interpretation of data, critical revision of the manuscript for intellectual content. YK: study concept and design, analysis and interpretation of data, critical revision of the manuscript for intellectual content. All authors contributed to the article and approved the submitted version.

## Conflict of Interest

The authors declare that the research was conducted in the absence of any commercial or financial relationships that could be construed as a potential conflict of interest.
