# Supplementary material for: Factors for Enhancement of Intracranial Atherosclerosis in High Resolution Vessel Wall MRI in Ischemic Stroke Patients
Source: Front Neurol. 2020 Jun 26;11:580. doi: 10.3389/fneur.2020.00580 (PMC7333774; doi:10.3389/fneur.2020.00580)
Supplement: Supplementary file 1 [file Data_Sheet_1.docx]

**Supplementary materials (online only)**

**Supplementary table 1: Odds ratio (95% confidence intervals) for enhancement of ICAS on VW-MRI by age and sex**

|  | Total cholesterol  per 1 mmol/L | Triglyceride  per 1 mmol/L | Low density lipoprotein cholesterol  per 1 mmol/L | Non-HDL  per 1 mmol/L | Apo (b) lipoprotein per 1 g/L | Apo (b)/Apo (a) ratio |
| --- | --- | --- | --- | --- | --- | --- |
| Age |  |  |  |  |  |  |
| < 57 yr | 4.637  (1.27 - 16.927) | 3.642  (1.033 - 12.838) | 3.559  (1.102 - 11.49) | 4.765  (1.322 - 17.168) | 2136.699  (6.688 - 682611.943) | 157.497  (1.729 - 14360.442) |
| ≥ 57 yr | 2.547  (1.064 - 6.093) | 1.763  (0.491 - 6.335) | 3.198  (1.01 - 10.121) | 2.567  (0.986 - 6.681) | 46.053  (1.18 - 1797.719) | 45.135  (0.34 - 5984.318) |
| Sex |  |  |  |  |  |  |
| Male | 2.505  (1.171 - 5.359) | 2.643  (0.925 - 7.55) | 2.349  (1.012 - 5.44) | 2.476  (1.139 - 5.382) | 66.436  (2.733 - 1615.178) | 38.926  (1.544 - 981.628) |
| Female | 7.258  (1.209 - 43.577) | 6.009  (0.455 - 79.34) | 12.137  (1.231 - 119.71) | 11.087  (1.289 - 95.368) | 19313.249  (4.001 - 93220729.39) | 1144433.563  (0.833 - 1000000) |

ICAS, Intracranial atherosclerosis; VW-MRI, High-resolution vessel wall MRI; HDL, High-density lipoprotein

**Supplementary table 2: Factors associated with high T1 signal or enhancement pattern**

|  | High T1 signal (-) (n = 22) | High T1 signal (+) (n = 6) | p | Non-eccentric enhancement (n = 2) | Eccentric enhancement (n = 26) | p |
| --- | --- | --- | --- | --- | --- | --- |
| Age, y | 61 (50 - 71) | 60.5 (48 - 71) | .0.844 | 55 (45 - 65) | 61 (50 - 71) | 0.6 |
| Male sex | 14 (63.6) | 4 (66.7) | 1 | 1 (50.0) | 17 (65.4) | 1 |
| Body mass index, kg/m^2^ | 24.5 (23.7 - 26.5) | 25.2 (21.5 - 26.9) | 0.93 | 26.1 (24.3 - 27.8) | 24.5 (23.2 - 26.5) | 0.6 |
| Hypertension | 15 (68.2) | 4 (66.7) | 1 | 1 (50.0) | 18 (69.2) | 1 |
| Diabetes | 5 (22.7) | 2 (33.3) | 0.62 | 0 (0.0) | 7 (26.9) | 1 |
| Hyperlipidemia | 7 (31.8) | 2 (33.3) | 1 | 0 (0.0) | 9 (34.6) | 1 |
| Metabolic syndrome | 0 (0.0) | 1 (16.7) | 0.21 | 0 (0.0) | 1 (3.8) | 1 |
| Smoker | 11 (50.0) | 4 (66.7) | 0.66 | 1 (50.0) | 14 (53.8) | 1 |
| Previous ischemic heart disease | 4 (18.2) | 0 (0.0) | 0.55 | 0 (0.0) | 4 (15.4) | 1 |
| Peripheral arterial occlusive diseases | 1 (4.5) | 0 (0.0) | 1 | 0 (0.0) | 1 (3.8) | 1 |
| Previous ischemic stroke | 4 (18.2) | 1 (16.7) | 1 | 0 (0.0) | 5 (19.2) | 1 |
| Prior statin use | 4 (18.2) | 1 (16.7) | 1 | 0 (0.0) | 5 (19.2) | 1 |
| Cerebral artery stenosis |  |  | 0.02 |  |  | 0.5 |
| Mild to moderate stenosis | 12 (54.5) | 0 (0.0) |  | 0 (0.0) | 12 (46.2) |  |
| Severe stenosis or occlusion | 10 (45.5) | 6 (100.0) |  | 2 (100.0) | 14 (53.8) |  |
| Laboratory findings |  |  |  |  |  |  |
| Total cholesterol, mmol/L | 5.3 (5.1 - 5.5) | 5.4 (5 - 5.9) | 0.61 | 5.3 (5.3 - 5.3) | 5.4 (5 - 5.7) | 0.7 |
| Triglyceride, mmol/L | 1.5 (1.1 - 2.6) | 1.7 (1.1 - 3.6) | 0.61 | 2.5 (2.1 - 2.9) | 1.5 (1.1 - 2.6) | 0.3 |
| High density lipoprotein cholesterol, mmol/L | 1.2 (1 - 1.4) | 1.2 (1 - 1.3) | 0.31 | 1 (0.8 - 1.1) | 1.3 (1 - 1.4) | 0.2 |
| Low density lipoprotein cholesterol, mmol/L | 3.2 (2.9 - 3.7) | 3.4 (2.3 - 3.8) | 0.96 | 3.3 (3.3 - 3.3) | 3.2 (2.5 - 3.8) | 0.8 |
| Non-HDL cholesterol, mmol/L | 4.0 (3.2 - 4.5) | 4.2 (4.1 - 4.5) | 0.42 | 4.3 (4.2 - 4.5) | 4 (3.2 - 4.5) | 0.4 |
| Apo (b) lipoprotein, g/L | 1.2 (1.2 - 1.4) | 1.2 (1.1 - 1.4) | 0.98 | 1.3 (1.2 - 1.4) | 1.2 (1.1 - 1.4) | 0.3 |
| Apo (a) lipoprotein, g/L | 1.4 (1.3 - 1.6) | 1.2 (1 - 1.5) | 0.25 | 1.2 (1.1 - 1.4) | 1.4 (1.2 - 1.6) | 0.3 |
| Apo (b)/(a) lipoprotein ratio | 0.8 (0.7 - 1.1) | 0.9 (0.9 - 1.0) | 0.31 | 1.1 (0.9 - 1.3) | 0.8 (0.7 - 1.1) | 0.2 |
| Blood Urea Nitrogen, mmol/L | 5.3 (4.2 - 7.1) | 6.4 (4.8 - 7.2) | 0.37 | 3.4 (3.3 - 3.5) | 5.5 (4.7 - 7.2) | 0 |
| Creatinine, µmol/L | 61.4 (49.5 - 72.5) | 77.4 (59.2 - 87.5) | 0.22 | 54.4 (43.3 - 65.4) | 64.1 (50.4 - 78.7) | 0.4 |
| Albumin, g/L | 42 (41 - 45) | 43 (41 - 44) | 0.76 | 43 (41 - 45) | 42.5 (41 - 44) | 0.9 |
| Protein level, g/L | 69 (66 - 73) | 70.5 (69 - 73) | 0.59 | 73 (66 - 80) | 69 (67 - 73) | 0.7 |
| Creatinine kinase, µkat/L | 1.4 (0.8 - 2.6) | 1.2 (1.1 - 1.4) | 0.82 | 1.6 (0.8 - 2.5) | 1.3 (1 - 2.6) | 0.8 |
| C-reactive protein, nmol/L | 11.4 (4.8 - 23.8) | 16.7 (8.6 - 23.8) | 0.43 | 7.6 (3.8 - 11.4) | 14.3 (5.7 - 23.8) | 0.4 |
| D-dimer, nmol/L | 599.6 (361.4 - 974.7) | 665.3 (443.6 - 821.4) | 0.65 | 470.9 (460 - 481.9) | 654.4 (394.3 - 974.7) | 0.5 |
| White blood cell count, 10^9^/L | 0 (0 - 0) | 0 (0 - 0) | 0.2 | 0 (0 - 0) | 0 (0 - 0) | 0.3 |
| Hemoglobin, g/L | 149 (138 - 163) | 139.5 (124 - 166) | 0.4 | 151.5 (146 - 157) | 147.5 (137 - 164) | 0.9 |
| Platelet count | 229.5 (213 - 247) | 273 (257 - 298) | 0.02 | 238.5 (221 - 256) | 239.5 (213 - 257) | 0.9 |
| HbA1c | 0.1 (0.1 - 0.1) | 0.1 (0.1 - 0.1) | 0.1 | 0.1 (0.1 - 0.1) | 0.1 (0.1 - 0.1) | 0.1 |
| Fasting glucose, mmol/L | 6.9 (5.7 - 8.3) | 7.5 (5.9 - 12) | 0.61 | 7.3 (6.3 - 8.3) | 6.9 (5.7 - 9.2) | 0.9 |
| Erythrocyte sedimentation ratio, mm/h | 17.5 (7 - 25) | 19 (9 - 33) | 0.7 | 10 (3 - 17) | 18 (8 - 32) | 0.3 |
| Fibrinogen level, µmol/L | 9 (7.1 - 10.6) | 9.1 (8.4 - 10.5) | 0.7 | 10.2 (9.4 - 11) | 9 (7.3 - 10.5) | 0.3 |

**Supplementary table 3: Factors associated with reconstruction index**

|  | r or median (IQR) | p |
| --- | --- | --- |
| Age, y | -0.032 | 0.871 |
| Sex |  | 0.332 |
| Male | 1.1 (1.0 - 1.2) |  |
| Female | 1.1 (1.0 - 1.1) |  |
| Body mass index, kg/m^2^ | -0.299 | 0.130 |
| Hypertension |  | 0.585 |
| N | 1.1 (1.0 - 1.2) |  |
| Y | 1.1 (1.0 - 1.1) |  |
| Diabetes |  | 0.611 |
| N | 1.1 (1.0 - 1.2) |  |
| Y | 1.1 (1.0 - 1.1) |  |
| Hyperlipidemia |  | 0.535 |
| N | 1.1 (1.0 - 1.2) |  |
| Y | 1.1 (1.0 - 1.1) |  |
| Metabolic syndrome |  | 0.454 |
| N | 1.1 (1.0 - 1.1) |  |
| Y | 1.1 ( 1.0 - 1.1) |  |
| Smoker |  | 0.646 |
| N | 1.1 (1.0 - 1.1) |  |
| Y | 1.1 (1.0 - 1.1) |  |
| Previous ischemic heart disease |  | 0.817 |
| N | 1.1 (1.0 - 1.1) |  |
| Y | 1.1 (1.0 - 1.1) |  |
| Peripheral arterial occlusive diseases |  |  |
| N |  |  |
| Y |  |  |
| Previous ischemic stroke |  | 0.238 |
| N | 1.1 (1.0 - 1.1) |  |
| Y | 1.1 (1.1 - 1.1) |  |
| Prior statin use |  | 0.928 |
| N | 1.1 (1.0 - 1.2) |  |
| Y | 1.1 (1.0 - 1.1) |  |
| Cerebral artery stenosis |  | 0.02 |
| No severe stenosis | 1.0 (1.0 - 1.1) |  |
| Severe stenosis (≥50%) or occlusion | 1.1 (1.1 - 1.2) |  |
| Laboratory findings |  |  |
| Total cholesterol, mmol/L | 0.334 | 0.083 |
| Triglyceride, mmol/L | 0.005 | 0.978 |
| High density lipoprotein cholesterol, mmol/L | 0.090 | 0.648 |
| Low density lipoprotein cholesterol, mmol/L | 0.315 | 0.102 |
| Non-HDL cholesterol, mmol/L | 0.243 | 0.212 |
| Apo (b) lipoprotein, g/L | 0.111 | 0.572 |
| Apo (a) lipoprotein, g/L | 0.125 | 0.527 |
| Apo (b)/(a) lipoprotein ratio | 0.076 | 0.702 |
| Blood Urea Nitrogen, mmol/L | -0.056 | 0.778 |
| Creatinine, µmol/L | 0.058 | 0.770 |
| Albumin, g/L | 0.236 | 0.227 |
| Protein level, g/L | 0.037 | 0.850 |
| Creatinine kinase, µkat/L | -0.241 | 0.216 |
| C-reactive protein, nmol/L | -0.010 | 0.959 |
| D-dimer, nmol/L | 0.144 | 0.464 |
| White blood cell count, 10^9^/L | 0.067 | 0.734 |
| Hemoglobin, g/L | 0.064 | 0.747 |
| Platelet count | 0.304 | 0.116 |
| HbA1c | -0.007 | 0.970 |
| Fasting glucose, mmol/L | -0.099 | 0.618 |
| Erythrocyte sedimentation ratio, mm/h | -0.201 | 0.304 |
| Fibrinogen level, µmol/L | -0.438 | 0.020 |
